# Indoor Air Quality Levels in Schools: Role of Student Activities and No Activities

**DOI:** 10.3390/ijerph17186695

**Published:** 2020-09-14

**Authors:** Gaetano Settimo, Luciana Indinnimeo, Marco Inglessis, Marco De Felice, Roberta Morlino, Annalisa di Coste, Alessandra Fratianni, Pasquale Avino

**Affiliations:** 1 Environment and Health Department, Italian National Institute of Health, viale Regina Elena 299, I-00185 Rome, Italy; gaetano.settimo@iss.it (G.S.); marco.inglessis@iss.it (M.I.); marco.defelice@iss.it (M.D.F.); roberta.morlino@iss.it (R.M.); 2 Department of Pediatrics and Child Neuropsychiatry, Policlinico Umberto I, University of Rome La Sapienza I, Viale Regina Elena 324, I-00161 Rome, Italy; luciana.indinnimeo@uniroma1.it (L.I.); dicoste.annalisa@gmail.com (A.d.C.); 3 Department of Agriculture, Environmental and Food Sciences, University of Molise, via F. De Sanctis, I-86100 Campobasso, Italy; alessandra.fratianni@unimol.it

**Keywords:** indoor air quality, school, student, CO_2_, VOCs, PM_2.5_, denuder, PCA, risk

## Abstract

This work describes a methodology for the definition of indoor air quality monitoring plans in schools and above all to improve the knowledge and evaluation of the indoor concentration levels of some chemical pollutants. The aim is to guide interventions to improve the health of students and exposed staff connected with the activities carried out there. The proposed methodology is based on the simultaneous study of chemical (indoor/outdoor PM_2.5_, NO_2_, CO_2_) and physical (temperature, humidity) parameters by means of automatic analyzers coupled with gaseous compounds (benzene, toluene, ethylbenzene, xylenes, formaldehyde and NO_2_) sampled by denuders. The important novelty is that all the data were collected daily in two different situations, i.e., during school activities and no-school activities, allowing us to evaluate the exposure of each student or person. The different behaviors of all the measured pollutants during the two different situations are reported and commented on. Finally, a statistical approach will show how the investigated compounds are distributed around the two components of combustion processes and photochemical reactions.

## 1. Introduction

Indoor air quality (IAQ) is an important determinant for the health of the general population, especially for susceptible population groups, such as children and adolescents who spend most of their time in confined spaces (domestic and school) [1,2]. IAQ is conditioned by both external and internal sources of pollution. The first derives from external pollutants that usually penetrate through the opening of the windows, whereas the internal sources can come from combustion processes (e.g., nitrogen dioxide (NO_2_)) or can be represented by construction materials, furniture and commonly used products for cleaning domestic environments (e.g., volatile organic compounds (VOCs)) [3,4]. The indoor environment therefore contributes significantly to the exposure to pollutants, many of which have a higher concentration indoors than outdoors [5]. Due to the high number of emission sources that can be identified in confined environments and the consequent high concentration of pollutants, indoor pollution is therefore considered an extremely complex and difficult to control form of pollution [6,7,8,9,10]. An increasing number of studies have confirmed that exposure to indoor pollutants causes a greater risk of respiratory disorders, probably consequent to a marked inflammation of the airways, which underlies oxidative stress mechanisms [11,12,13,14,15,16,17,18,19,20]. In addition, indoor allergens are the main cause of sensitization and exacerbation in asthmatic subjects [21]. Among the sources of pollution, indoor allergens are one of the main causes of sensitization and of exacerbation and triggering of acute asthma attacks [22,23,24,25,26].

In recent years, the European Union (EU) has promoted and funded important indoor pollution projects in schools to improve knowledge in this area and encourage the reduction of cases of respiratory diseases and childhood asthma in Europe. Simultaneously, different Italian groups deal with this issue. The Health Effects of the School Environment (HESE) study was the first important European project on health problems related to pollution of the school environment [27], whereas the Schools Indoor Pollution and Health Observatory Network in Europe (SINPHONIE) [28] and School Environment and Respiratory Health of Children (SEARCH) [29] projects were part of the European Action Plan on Environment and Health 2004–2012. Finally, the two-year project Indoor and Outdoor Air quality and Respiratory Health in Malta and Sicily (RESPIRA) study was developed within the Italy-Malta Cross-border Cooperation Program [30]. European studies highlight the close relationship between exposure to indoor pollutants and the appearance of respiratory and allergic symptoms in childhood. The symptomatology described can significantly compromise the quality of life of children and their school performance. In line with the European initiative, the Italian Ministry of Health funded a project “Exposure to indoor pollutants: guidelines for assessing risk factors in the school and defining measures to protect respiratory health of schoolchildren and teenagers (Indoor-School)” [31]. The project was aimed to study the exposure of pupils and school staff (of primary and secondary schools) to indoor pollutants and assess the relationships between this exposure and the effects on health, with the aim of improving epidemiological knowledge in this area and facilitating the application of the “Guidelines for the prevention of indoor risk factors for allergies and asthma in schools” by the World Health Organization (WHO) [32].

In the frame of the Indoor-School project, a task was dedicated to highlight differences in IAQ in the presence and absence of students. This task is apparently not really important but is becomes fundamental for a correct student exposure risk assessment. In fact, the studies on the indoor school are basically oriented to perform measurements of gaseous/aerosol pollutants every day or during school activities only [33]. In this paper the approach is different: the measurements were performed taking into account the presence or absence of school activity both for showing the relative contribution to the IAQ and for studying the inhalation intake by students.

## 2. Experiments

### 2.1. Operating Protocol

As part of the Indoor-School project, field surveys were carried out for the determination of selected chemical pollutants and the main microclimatic parameters with the aim of determining the levels of these pollutants in the selected school buildings. The pollutants for which the concentration in this matrix was determined are volatile organic compounds (VOCs) such as benzene, toluene, xylenes, ethylbenzene, aldehydes, PM_2.5_ and NO_2_. The CO_2_ concentration, the percentage relative humidity, the temperature and the speed of the air were also determined, thus increasing the information of the classrooms. For this purpose, the following methods and equipment were used:

1. VOCs by sampling using passive Radiello^®^ (AMS Analitica, Pesaro, Italy) samplers and quantitative gas chromatographic determination;
2. NO_2_ by sampling using passive Radiello^®^ samplers and gas chromatography determination;
3. Particulate matter (PM_2.5_) by means of direct reading analyzers (mod. DustTrak) (TSI, Shoreview, MN, USA) [34];
4. CO_2_, humidity, temperature, and air speed by means of a q-track analyzer (TSI).

Two DustTrak instruments and one Q-Trak instrument were used for the measurements. Both the DustTraks were used at a sampling rate of 3 L min^−1^ (it is not possible to change the sampling rate), and the Q-Trak was used according to the parameters reported in the relative manual.

**Figure 1:**
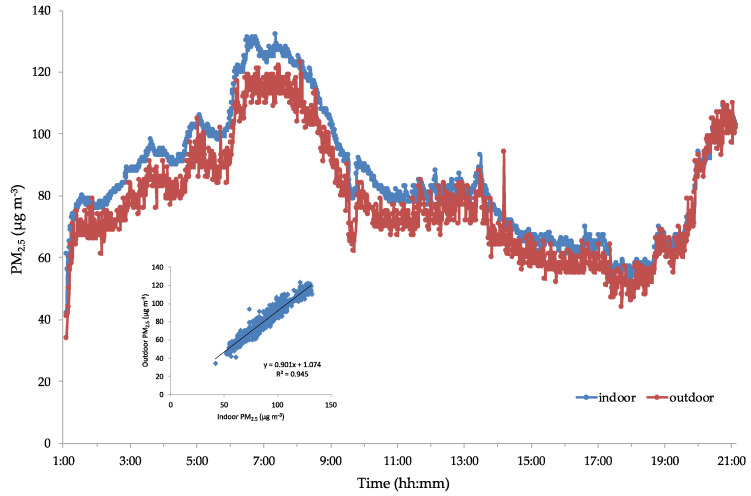
Field-comparison between two DustTrak instruments used for comparing indoor and outdoor measurements. The inner plot represents the correlation equation between the two datasets.

Before each sampling campaign, a task was dedicated to compare the instruments used for performing the indoor and outdoor measurements. Figure 1 illustrates this comparison: there is high correlation (R^2^ 0.945) between the measurements.

Subsequently, external ambient air samples were taken simultaneously at all sites to estimate their contribution. The whole study was performed in winter. All data were collected and analyzed in terms of mean and standard deviation (s.d.) using usual mathematical software: further, mean and s.d. were also measured during school activities and no-school activities.

### 2.2. School Buildings to Be Monitored

Before studying the main chemical pollutants in indoor air, it was very useful to collect information and characteristic data of the environments that are part of the school building (e.g., times and frequency of use of the classrooms and other spaces). Eight school buildings were selected for the study (three classes for each school). All the schools were selected in Rome. We paid particular attention to the selection: in fact, they were chosen in the downtown area, in areas with a high density of traffic, and with other buildings close to them. Further, the schools are in historical buildings where the ventilation is only provided by large windows in each classroom. This meant the investigated scenarios were quite similar. In each classroom, surveys were carried out in one or two sampling points, deemed suitable for estimating indoor exposure. The most suitable withdrawal point was considered the center of the classroom or next to the chair if away from windows or ventilation/heating systems. The same point was used for the measurement of CO_2_, humidity, temperature, and air speed. In addition, the chosen pick-up points were the same in all classrooms of the eight schools. Finally, the samplers were positioned approximately 1–1.5 m above the ground, and not less than 1 m away from the wall.

### 2.3. Classroom Withdrawals for VOC and NO_2_

Three different Radiello^®^ were used with picking times divided as follows [35,36,37]:

1. 1 + 1 Radiello^®^ must cover the timetable of school activities (i.e., 7:00–14:00),
2. 1 + 1 Radiello^®^ must cover the non-activity time (i.e., 14:00–7:00),
3. 1 + 1 Radiello^®^ must cover the whole day.

At the same time, an additional external withdrawal was performed, to estimate the contribution of the outdoor exposure (two/three withdrawal points) of 24 h.

In the study, the VOCs of greatest interest from the sanitary point of view were identified and considered (benzene, toluene, ethylbenzene, *o*-*m*-*p*-xylenes, formaldehyde).

The Radiello^®^ radial diffusion sampler consists of a diffusion tube (cartridge) that uses the physical diffusion (sampling) process of pollutants, and a cylindrical cartridge in stainless steel mesh containing activated carbon or another type of adsorbent. During the sampling, the cartridge was placed inside a microporous cylindrical diffusive body and mounted on a support. These samplers were used for VOC and NO_2_ sampling at the sampling sites for a period of six consecutive days.

The Italian situation regarding indoor pollution has moved towards a progressive adaptation to European standards with the implementation by UNI of the standards of the European Standardization Committee (CEN) EN 838/95 [38]. The aforementioned standards contain indications for carrying out sampling of COVs, EN ISO 16000–5: Sampling strategy for volatile organic compounds (VOC), reporting specific measurement techniques.

### 2.4. The Chemiometric Approach

Starting with the data obtained by the denuders, the authors applied a chemometric approach for evaluating possible correlations among the different situations. Tanagra open-source software [39] was used by means of the centroid merge method and the Euclidean distance as a proximity measure [40,41].

## 3. Results

One of the main objectives of this project was the definition of the IAQ parameters during school performances in the areas and environments selected for the study. In particular, environmental data inside classrooms were simultaneously measured, including temperature, relative humidity, carbon dioxide (CO_2_), and particulate matter ≤ 2.5 μm (PM_2.5_), according to the reference method UNI EN 14907. On the other hand, benzene (C_6_H_6_), toluene (C_7_H_8_), xylenes (as sum of *ortho*-, *meta*- and *para*-xylene) (C_8_H_10_), ethylbenzene (C_8_H_10_), formaldehyde (HCHO) and nitrogen dioxide (NO_2_) were determined outside the school.

Table 1 shows the average daily levels measured in different classrooms in eight schools located in downtown Rome. As it can be seen, even if the sampling campaigns were carried out at the same time, the microclimate among the different schools was different, meaning possible differences in the IAQ interpretation. In particular, the temperature ranged between 17.9 and 25.1 °C with variability (as coefficient of variation, cv%) between 2.7 and 15.0%, whereas the relative humidity was between 32.8 and 53.9% with variability from 2.5 and 17.8%. This occurrence could be coupled with the column showing the carbon dioxide concentrations: the levels ranged from 653 to 1352 ppm with cv% between 8.3 and 70.9%. This large CO_2_ variability during the day is due to possible non-school activities in the afternoon, when students are not in, but different events could happen (e.g., gym, theater, meetings volleyball), typical occurrences in Italian schools. Finally, indoor PM_2.5_, ranging between 18.4 and 56.2 µg m^−3^, was determined to understand the effect of the air changes and the presence of students on the relative levels, whereas outdoor PM_2.5_, from 11.8 to 79.4 µg m^−3^, was an index of the external/internal pollution sources.

Starting from these preliminary considerations, the authors focused their attention on the levels of PM_2.5_ and CO_2_ during school activity and no-school activity. Table 2 reports these data. The levels of two such pollutants determined during school activity (from 7:00 to 14:00, i.e., during the activities related to the presence of the students) were basically higher that those determined in the other period (14:00–7:00) when other or no activities occured, except in two cases (school #D and school #G) where the differences between PM_2.5_ levels are very close (39.1 vs. 44.9 µg m^−3^ and 46.7 vs. 48.6 µg m^−3^) and CO_2_ is what is expected (1627 vs. 918 ppm).

Finally, Table 3 shows the levels of benzene, toluene, xylenes, ethylbenzene, formaldehyde and nitrogen dioxide after sampling with denuders and analysis in the laboratory. The passive samplers provide averaged concentration values of the pollutants over one or more days and therefore allow us to evaluate a large period of time and to take into account any changes related to the work cycles and civil activities that take place on the site in question [42,43,44]. Indoor levels of gaseous pollutants, such as benzene and ethylbenzene, are below the limit of detection (LOD) of the methodology used, whereas relevant formaldehyde concentrations were determined in both situations, especially in two schools during the no-school activity.

## 4. Discussion

### 4.1. IAQ: Gaseous/Airborne Pollutants and Denuders

IAQ is strongly influenced by furnishings as possible sources of pollutants. Another important aspect for the IAQ evaluation concerns the management of cleaning [45,46]. Guidelines establish that it is appropriate to carry out the cleaning operations in the absence of the students and at least a few hours before their entry into the classroom.

This paper would like to propose a methodology for identifying possible sources of air pollution during use and in the presence of students and staff, determining the concentration levels of some pollutants in different classrooms, and comparing the different concentrations with the guide values, as well as checking the correct functioning of the air conditioning technology and specific air exchanges.

According to the data, although less than half of the schools had operating protocols indicating the procedures to be followed when cleaning, these were carried out mainly after the lessons. The sources present in the indoor environments of the eight school buildings investigated, together with the type of activity carried out by the students, entailed the release of various types of chemical pollutants into the air.

**Figure 2:**
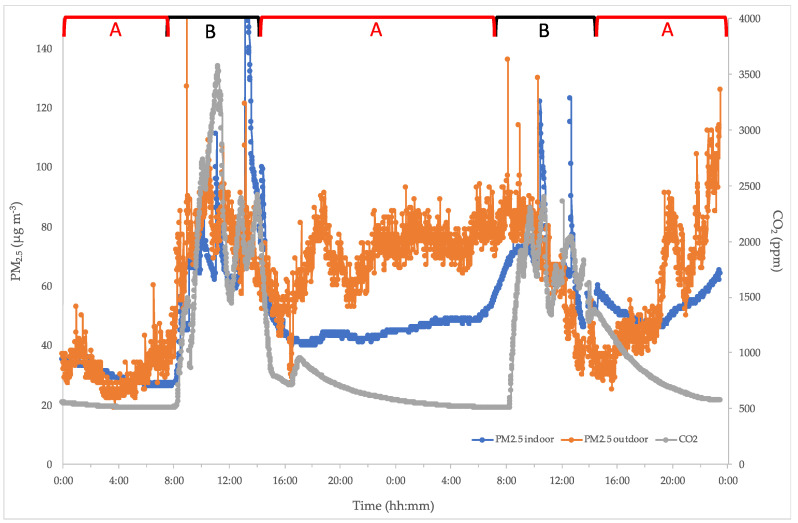
Indoor/outdoor PM_2.5_ and CO_2_ levels during 48 h-continuous measures. **A**: no school activity (14:00–7:00); **B**: school activity (7:00–14:00).

Figure 2 shows the typical indoor/outdoor PM_2.5_ and CO_2_ trends in two consecutive days in schools where only school activities are present.

As can be seen, the indoor PM_2.5_ and CO_2_ rapidly increase their levels as soon as the activities begin, e.g., administrative staff from 7:00 and students from 8:00. In this period, a strict correlation between PM_2.5_ and CO_2_ is detected in all schools where no other activities are present. This correlation is around 0.75 but, in some cases, it even reaches 0.86. Further, another parameter in regards to the effects of outdoor PM_2.5_ is opening windows. The effect of this is to temporarily dilute the pollutants and increase the IAQ (recorded in Figure 2 as partial decreases in CO_2_). At the end of school activities, i.e., the end of both lessons and work by administrative staff (14:00), a clear decrease of indoor PM_2.5_ and CO_2_ is detected. A CO_2_ peak can be revealed around 16:00 along with a low PM_2.5_ increase. This occurrence, due to the cleaning procedures performed by the staff, is carried out without paying attention to opening the windows; the effect is to increase the CO_2_ levels in that moment but, mainly, to increase the background level of indoor PM_2.5_ that will persist until the next day. This means that the effects of cleaning processes not performed according to safety procedures can create risks for the health of exposed people, even after hours. Furthermore, as will be shown later, it is also necessary to consider the persistence and the relative effect of both the VOCs emitted by the products used for cleaning or even the cigarette smoke by the cleaning operators.

**Figure 3:**
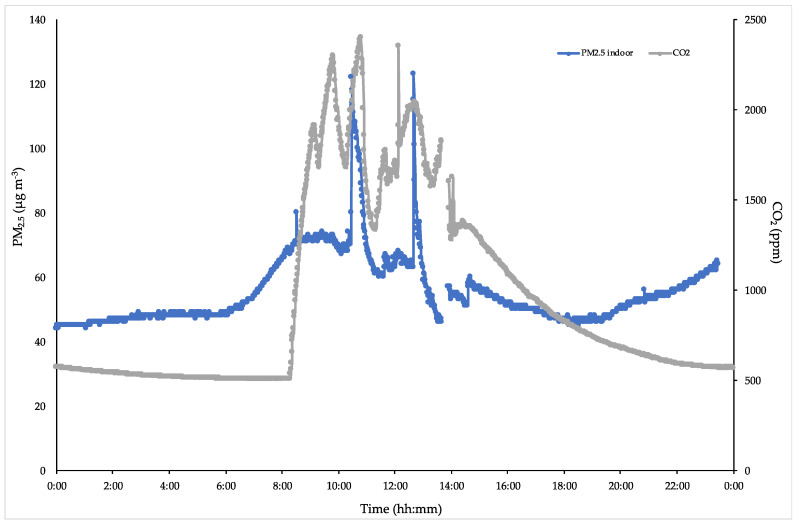
Typical indoor PM_2.5_ and CO_2_ behaviors in classes where only school activities are daily performed.

Figure 3 shows a highlight of the previous figure. It is evident that the start of activities as well as the effect of air exchange at the end of every lesson is evidenced by hourly CO_2_ decreases, whereas PM_2.5_ constantly increases its values up to an almost steady state, with some relevant peaks due to occasional events (opening windows). This typical behavior, occurring in every class when no activities are performed after the lessons, allow us to draw a baseline level of these two pollutants in any situations and estimate the risk assessment for the exposed personnel (students, teachers, administrative staff, etc.).

**Figure 4:**
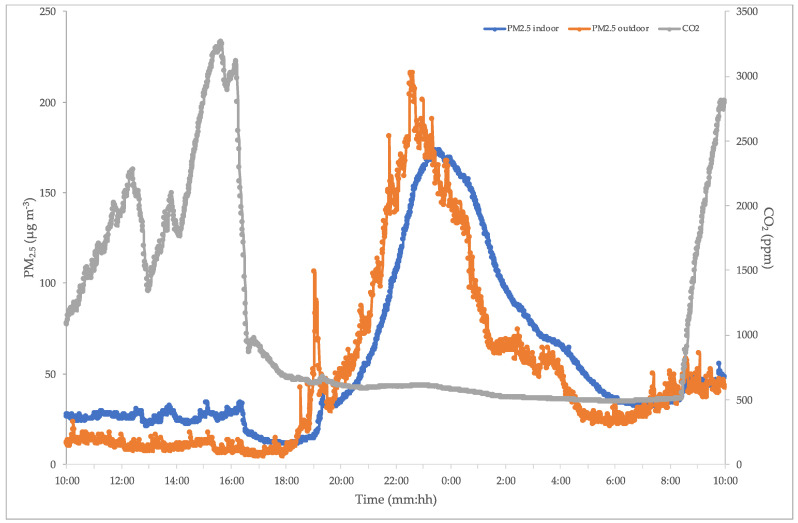
PM_2.5_ and CO_2_ levels in a class during afternoon/evening events.

The schools host daily afternoon events from 15:00 until 19:00 or 20:00 (some events are also at night). These events could be volleyball/basketball gym, theater, or dance school, and often include a lot of people. These situations commonly occur in Italy, especially in schools present in big urban cities such as Rome. Because of this, we investigated the pollution during such occurrences. Figure 4 shows the effects of an intense afternoon/evening activity on the indoor PM_2.5_ and CO_2_.

The most interesting issue is related to the occurrence after the end of school-activities. As can be seen, two different situations can be detected: from 14:00 to 17:00, indoor and outdoor PM_2.5_ show almost the same levels, which are quite low without relevant peaks. During this period, indoor PM is higher than outdoor, as can be expected, whereas CO_2_ reaches high levels (up to 3500 ppm) from 14:00 to 17:00 due to presence of students in the gyms. The authors would like to remember that the average residential indoor CO_2_ level is recommended to be 1000 ppm [47] but in some countries it can reach levels of 1200–1250 ppm [48,49], 1500 ppm [50], or up to 3500 ppm [51]. From 19:00 this occurrence is completely changed, the behaviors are overturned: CO_2_ becomes stationary, almost constant, around 500 ppm, whereas indoor/outdoor PM_2.5_ increases up to 230 µg m^−3^. What accounts for these findings? A retrospective analysis shows a close relationship between indoor and outdoor PM_2.5_ and low CO_2_ level: this suggests the presence of an event where the air exchange is really important (low CO_2_) and simultaneously there is high airflow from outside (similar PM values). In fact, the School White Night occurred with a large participation of people and high air remixing.

The approach followed in this study shows a few limitations, essentially due to the sampling procedure. In fact, sampling carried out in the outdoor environment should follow the same protocol as the indoor sampling (showing a difference between the time of activities and no activities), to be able to consider the influence of the outdoor environment on the indoor environment.

During the measurement campaigns, denuders played an important role. Gaseous pollutants were determined following the sampling and the laboratory analysis. As reported in Table 3, denuders allowed us to take information on some indoor/outdoor gaseous pollutants such as benzene, toluene, xylenes, ethylbenzene, formaldehyde and NO_2_. Few papers have dealt with the pollutant determination by denuders in schools [52,53,54,55,56] but no papers address the relative evaluation differentiating between school activity and no-school activity. Once again, this is the main novelty of this paper. The authors would like to underline that this difference could be useful for determining the risk of personal exposure of each student.

In this case, the scenario is quite different than the one shown for PM_2.5_ and CO_2_, except in very few cases, the levels determined during no-school activities, i.e., between 14:00 to 7:00, are higher than those determined during school activities, i.e., 7:00–14:00. These high levels are essentially due to the cleaning procedures occurring in the afternoon. A recent paper demonstrated the levels of ultrafine particles produced by floor cleaning products [46]. It is reasonable to think that the different cleaning products used in the different schools are responsible for the high PM_2.5_ levels as well as for the high levels of some gaseous pollutants. Further, the formaldehyde levels are quite interesting: although they could appear high, they are below the WHO guideline values [9] (100 µg m^−3^). Its levels were above the guideline value (113.6, 115.8 and 165.2 µg m^−3^) only in three situations (two classes in school #D and 1 in school #F, respectively), due to smoking by cleaning staff. An isolated analysis for determining acetaldehyde in the three situations confirmed the theory [57]. The outdoor determinations confirmed some issues. For instance, the ratio between toluene/benzene, which is 3–5 in urban air, verifies the hypothesis that no other sources of these pollutants, except the autovehicular emissions, are present in the investigated areas [58,59]. Ethylbenzene is always below the LOD, whereas the xylenes are in line with previous determinations [60].

### 4.2. A Chemometric Approach

**Figure 5:**
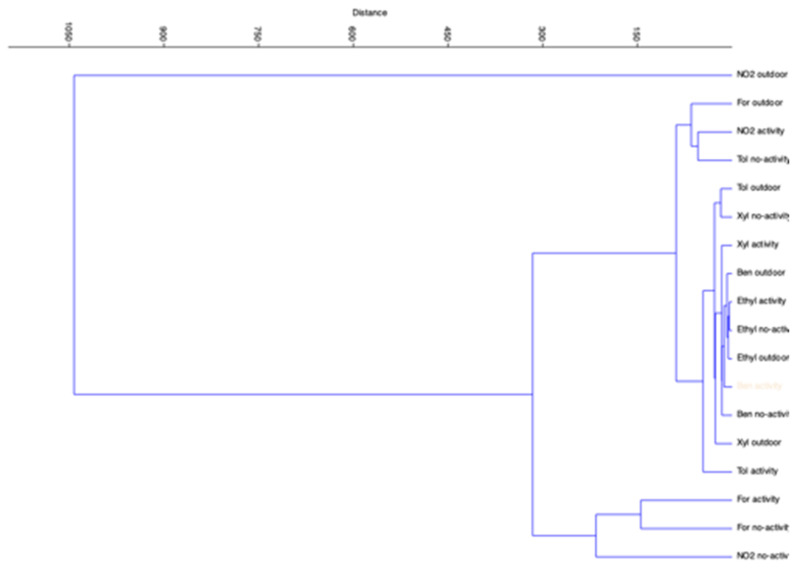
Cluster analysis related to compounds sampled in different situations in the eight schools.

Cluster analysis (CA) and principal component analysis (PCA) showed similarities among the data. In showing these similarities, we first used CA (Figure 5) for understanding how the dataset can be divided. The data are in five clusters:

1. cluster #1 is composed of three elements: formaldehyde outdoor, NO_2_ indoor during activity and toluene indoor during no-activity;
2. cluster #2 is composed of three elements: formaldehyde indoor during activity, formaldehyde indoor during no-activity, NO_2_ indoor during no-activity;
3. cluster #3 is composed of four elements: xylenes outdoor, xylenes indoor during no-activity, toluene outdoor, toluene indoor during activity.
4. cluster #4 is composed of seven elements: benzene outdoor, benzene indoor during activity, benzene indoor during no-activity, xylenes indoor during activity, ethylbenzene outdoor, ethylbenzene indoor during activity, ethylbenzene indoor during no-activity.
5. cluster #5 is composed of one element: NO_2_ outdoor.

**Figure 6:**
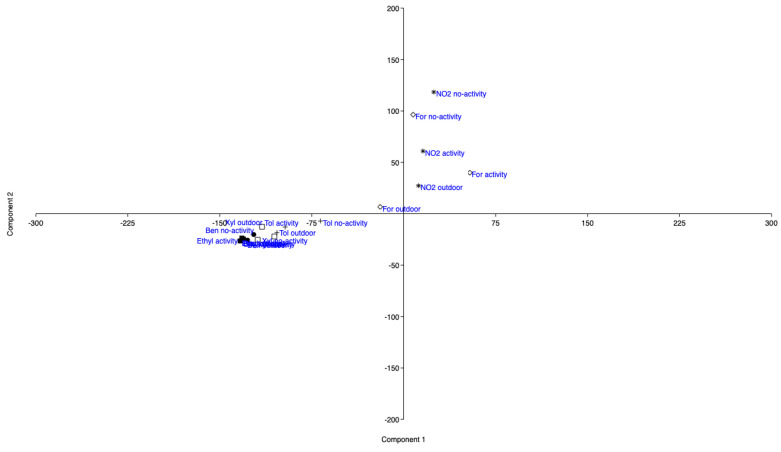
Principal component analysis (PCA) applied to the overall data. Component 1: compounds from photochemical activity; Component 2: compounds from combustion processes.

CA actually showed the presence of five clusters: for a better understanding of the representation, the authors would like to show the PCA. The first information regarding Component 1 and Component 2 is able to explain 92.3% of the overall data. In particular, Component 1 (i.e., a component formed by NO_2_ and formaldehyde determined indoor/outdoor) is related to compounds associated with photochemical reactions, whereas Component 2 (i.e., a component formed by toluene, benzene, ethylbenzene and xylenes determined indoor/outdoor) is related to compounds connected to combustion processes. As can be seen in Figure 6, the levels of NO_2_ and formaldehyde, both outdoor and during activity/no-activity, are distributed along Component 1, meaning that in such situations these compounds are predominant, whereas benzene, toluene, ethylbenzene and xylenes are relevant along Component 2, as expected.

## 5. Conclusions

The pursuit of improving IAQ in schools translates into a significant lifetime benefit for the health of students, teaching staff, technical and administrative staff. This paper would like to highlight the importance of some issues in the IAQ in schools:

1. to understand the close relationship between the school environment, health and indoor air pollution in the heterogeneous school context, through the acquisition of data on chemical pollutants;
2. to stimulate the correct choice and use of energy efficient processes;
3. to understand the need for regular air exchange;
4. to modernize classrooms, specialized didactic laboratories, gyms, offices, etc.;
5. to provide furnishings that are increasingly suitable for teaching;
6. to choose educational and consumer materials taking into account the emission levels of pollutants of the individual materials.

IAQ takes on particular significance and importance, both for the vulnerabilities of the subjects (e.g., students and workers, some with more or less complex diversified susceptibility and disability, or with respiratory, asthmatic and allergic diseases, or alteration of the immune system, etc.), and for the high residence times. In general, the environments and school spaces represent, after the home environment, the places where students spend more time, on average about 6–8 h a day for at least five days a week for nine months of the year, whereas for teachers, staff and administrative staff, it can be extended for shorter or longer periods.

For all these reasons, a series of new strategies, particularly appropriate and organized interventions, must be set up, which depend on many factors that cannot be limited to individual items (e.g., electrical, water, seismic, fire, architectural, or energy efficiency), without including the IAQ improvement among the interventions or priorities. All these adjustment interventions contribute significantly to the IAQ and the health of students and all staff.

## Figures and Tables

**Table 1:** Average daily levels of some physical-chemical parameters along with standard deviation ($\bar{x}$ + s.d.) and coefficient of variation (cv% ^1^) simultaneously determined during the entire measurement campaign in eight schools in downtown Rome.

Site	Temperature (°C)	Humidity (%)	PM_2.5_ Outdoor (µg m^−3^)	PM_2.5_ Indoor (µg m^−3^)	CO_2_ (ppm)
School #A	18.9 ± 2.2 (11.4)	53.1 ± 1.7 (3.1)	13.9 ± 12.8 (91.9)	18.7 ± 6.8 (36.2)	1352 ± 940 (70.9)
School #B	25.1 ± 0.9 (3.5)	53.9 ± 3.3 (6.2)	22.0 ± 7.8 (35.3)	25.5 ± 10.3 (40.3)	774 ± 352 (45.5)
School #C	17.9 ± 2.0 (11.1)	51.9 ± 4.8 (9.3)	11.8 ± 7.3 (62.2)	25.6 ± 17.2 (67.1)	1086 ± 756 (69.6)
School #D	19.2 ± 2.0 (10.3)	43.2 ± 5.3 (12.3)	30.0 ± 35.1 (116.9)	38.6 ± 29.9 (77.5)	1008 ± 691 (68.6)
School #E	22.7 ± 0.8 (3.5)	49.9 ± 6.4 (17.8)	15.2 ± 8.4 (55.4)	18.4 ± 19.9 (108.3)	684 ± 336 (49.0)
School #F	21.8 ± 2.2 (15.0)	44.7 ± 6.7 (15.0)	79.4 ± 62.8 (79.1)	56.2 ± 42.3 (75.3)	851 ± 571 (67.0)
School #G	22.7 ± 0.6 (2.7)	43.4 ± 1.1 (2.5)	61.7 ± 35.4 (57.4)	52.5 ± 23.2 (44.1)	653 ± 54 (8.3)
School #H	22.4 ± 4.1 (12.5)	32.8 ± 4.1 (12.5)	78.5 ± 44.5 (56.7)	51.0 ± 20.9 (40.9)	943 ± 561 (12.5)

^1^ cv% is reported in bracket.

**Table 2:** Daily levels of indoor PM_2.5_ (µg m^−3^) and CO_2_ (ppm), respectively, in eight schools in downtown Rome.

Site/Activity ^1^	x + s.d. ^2^	Min–Max	cv%	75th Perc.	95th Perc.	99th Perc.
School #A						
PM_2.5_ activity	18.3 ± 5.8	10.0–57.0	31.6	21.0	27.8	38.8
PM_2.5_ no activity	17.1 ± 4.0	13.0–34.0	23.1	18.0	27.0	31.0
CO_2_ activity	2386 ± 480	1401–3022	20.1	2759	2942	2993
CO_2_ no activity	599 ± 189	464–1653	31.5	644	1041	1171
School #B						
PM_2.5_ activity	31.0 ± 2.8	29.0–33.0	9.1	32.0	32.8	33.0
PM_2.5_ no activity	23.5 ± 5.5	16.0–101.0	23.2	26.0	30.0	42.8
CO_2_ activity	1380 ± 207	1233–1526	15.0	1453	1511	1523
CO_2_ no activity	606 ± 161	462–1218	26.6	653	1032	1182
School #C						
PM_2.5_ activity	23.4 ± 20.6	9.0–312.0	88.3	22.0	53.0	82.2
PM_2.5_ no activity	12.7 ± 17.5	6.0–324.0	137.6	11.0	32.0	64.1
CO_2_ activity	2021 ± 667	486–3192	33.0	2595	3033	3170
CO_2_ no activity	661 ± 325	466–2363	49.2	655	1354	2210
School #D						
PM_2.5_ activity	39.1 ± 7.0	30.0–74.0	18.0	45.0	50.0	57.0
PM_2.5_ no activity	44.9 ± 14.3	26.0–203.0	31.8	51.0	62.0	97.8
CO_2_ activity	1627 ± 806	487–2915	49.6	2275	2791	2888
CO_2_ no activity	918 ± 578	489–2833	62.9	1174	2351	2752
School #E						
PM_2.5_ activity	20.8 ± 5.7	10.0–38.0	27.5	23.0	32.0	35.0
PM_2.5_ no activity	16.3 ± 2.0	11.0–46.0	33.0	17.0	19.0	22.0
CO_2_ activity	674 ± 222	494–1316	12.3	710	1218	1308
CO_2_ no activity	535 ± 71	479–823	13.2	577	691	753
School #F						
PM_2.5_ activity	120.9 ± 11.2	101.0–156.0	9.3	126.0	143.0	152.0
PM_2.5_ no activity	60.7 ± 18.2	34.0–135.0	30.0	74.0	89.4	110.9
CO_2_ activity	1539 ± 606	521–2298	39.4	2079	2216	2279
CO_2_ no activity	678 ± 421	469–2049	62.1	537	1735	2004
School #G						
PM_2.5_ activity	46.7 ± 24.8	33.0–66.0	24.8	59.0	64.0	66.0
PM_2.5_ no activity	48.6 ± 24.4	18.0–118.0	50.2	67.0	94.2	101.8
CO_2_ activity	651 ± 55	564–845	8.5	688	754	806
CO_2_ no activity	N/A ^3^					
School #H						
PM_2.5_ activity	82.3 ± 41.2	59.0–411.0	50.1	79.5	149.6	264.5
PM_2.5_ no activity	46.8 ± 8.5	40.0–100.0	18.3	48.0	56.1	90.8
CO_2_ activity	2206 ± 633	858–3564	28.7	2645	3360	3515
CO_2_ no activity	697 ± 302	503–2410	43.3	751	1079	2286

^1^ school activity: 7.00–14.00; no school activity: 14.00–7.00. ^2^ s.d.: standard deviation. ^3^ N/A: not available.

**Table 3:** Indoor and outdoor levels (µg m^−3^) of gaseous pollutants sampled by denuders in eight schools in downtown Rome.

**Pollutant ^1^**	**School #A**				**School #B**				**School #C**				**School #D**			
**Class**	**1^ ^3^**	**2^**	**3^**		**5^**	**5^**	**4^**		**1^**	**2^**	**2^**		**3^**	**4^**	**5^**	
Benzene																
OA ^2^				2.6				1.1				0.98				1.5
A	<0.02	<0.02	<0.02		0.32	0.43	0.33		<0.02	<0.02	<0.02		<0.02	<0.02	<0.02	
NA	<0.02	0.8	3.1		0.60	<0.02	3.1		<0.02	1.8	6.3		<0.02	2.7	<0.02	
Toluene																
OA				7.9				6.6				8.6				9.0
A	7.7	6.4	8.2		7.7	7.4	8.2		9.2	7.1	6.7		8.9	5.8	9.4	
NA	13.1	4.6	19.3		13.4	5.3	19.4		14.0	6.9	23.1		18.3	7.0	<0.02	
Xylenes																
OA				2.6				3.2				2.9				2.5
A	2.2	2.1	4.6		2.7	1.9	5.1		3.1	4.6	4.9		3.4	4.3	6.0	
NA	3.9	4.1	8.8		4.5	5.0	7.6		5.2	7.9	9.7		4.6	6.8	<0.02	
Ethylbenzene																
OA				<0.02				<0.02				<0.02				<0.02
A	<0.02	<0.02	<0.02		<0.02	<0.02	<0.02		<0.02	<0.02	<0.02		<0.02	<0.02	<0.02	
NA	0.4	0.5	<0.02		0.4	0.5	<0.02		0.9	1.6	<0.02		2.1	1.6	<0.02	
Formaldehyde																
OA				39.4				21.1				24.3				23.2
A	76.6	62.5	63.2		83.7	82.9	95.6		82.6	65.6	57.4		84.8	81.8	92.4	
NA	38.5	38.3	37.4		46.3	55.3	59.1		83.7	40.8	51.7		113.6	115.8		
Nitrogen dioxide																
OA				254				245				266				99
A	24	12	12		12	14	12		13	12	12		27	31	27	
NA	49	42	35		58	70	50		27	61	68		35	54	29	
**Pollutant ^1^**	**School #E**				**School #F**				**School #G**				**School #H**			
**Class**	**1^**	**2^**	**3^**		**1^**	**2^**	**2^**		**1^**	**2^**	**2^**		**1^**	**2^**	**3^**	
Benzene																
OA				2.8				<0.02				1.0				1.2
A	<0.02	<0.02	1.2		<0.02	<0.02	<0.02		4.2	<0.02	<0.02		<0.02	<0.02	<0.02	
NA	5.1	5.2	5.3		<0.02	0.9	2.7		6.5	5.6	8.1		0.3	1.5	4.4	
Toluene																
OA				10.1				3.8				4.4				4.1
A	7.7	6.3	39.2		4.6	5.5	4.6		15.1	<0.02	13.5		6.1	6.4	5.8	
NA	19.4	20.1	20.3		13.1	7.0	17.4		20.2	18.0	26.3		17.0	6.8	18.4	
Xylenes																
OA				2.2				2.7				16				2.3
A	<0.02	<0.02	<0.02		3.2	2.6	4.1		4.0	<0.02	<0.02		2.9	3.0	5.2	
NA	4.2	4.4	4.6		3.8	6.0	9.3		5.0	4.2	5.7		4.9	7.3	8.4	
Ethylbenzene																
OA				<0.02				<0.02				3.5				<0.02
A	<0.02	<0.02	<0.02		<0.02	<0.02	0.3		<0.02	<0.02	<0.02		<0.02	<0.02	<0.02	
NA	<0.02	<0.02	<0.02		0.9	1.2	0.6		2.1	<0.02	<0.02		0.7	1.0	<0.02	
Formaldehyde																
OA				27.5				18.7				33.5				15.4
A	55.0	52.5	51.9		90.9	82.8	43.0		34.5	70.6	81.3		64.8	70.5	72.1	
NA	36.6	38.2	35.8			165.2	40.5		44.6	45.3	42.5		51.3	62.6	56.3	
Nitrogen dioxide																
OA				133				226				143				344
A	36	29	33		14	22	19		19	15	13		15	12	10	
NA	73	55	67		74	96	136		145	98	95		49	35	35	

^1^ activity: 7:00–14:00; no-activity: 14:00–7:00; ^2^ OA: outdoor; A: activity; NA: no-activity; ^3^ 1^,2^,3^,4^,5^: the class level in the Italian schools (different sections).
